# Supplementary material for: Clustering of Dietary Patterns Associated with Health-Related Quality of Life in Spanish Children and Adolescents
Source: Nutrients. 2024 Jul 18;16(14):2308. doi: 10.3390/nu16142308 (PMC11280478; doi:10.3390/nu16142308)
Supplement: Supplementary file 1 [file nutrients-16-02308-s001.zip › nutrients-3090733-supplementary.pdf]

Supplementary material

Table S1. Dietary patterns scores of each cluster established.

Food item	Cluster 1 (the unhealthiest)	Cluster 2 (the moderately healthy)	Cluster 3 (the healthiest)
Fruits (z score)	-0.2 (1.1)	-0.4 (1.1)	0.4 (0.7)
Meat (z score)	-0.2 (1.0)	0.3 (1.0)	-0.3 (0.9)
Eggs (z score)	0.0 (1.1)	0.2 (1.1)	-0.2 (0.9)
Fish (z score)	0.0 (1.0)	-0.1 (1.1)	0.1 (0.9)
Pasta, rice, potatoes (z score)	-0.2 (1.0)	0.2 (1.0)	-0.1 (1.0)
Bread, cereals (z score)	-2.2 (2.2)	0.2 (0.3)	0.2 (0.3)
Vegetables (z score)	-0.2 (1.1)	-0.2 (1.0)	0.2 (0.9)
Pulses (z score)	0.1 (1.0)	0.3 (1.0)	-0.2 (0.9)
Processed meat (z score)	-0.4 (1.0)	0.4 (0.9)	-0.3 (1.0)
Dairies (z score)	-2.0 (2.5)	0.2 (0.3)	0.2 (0.2)
Cookies, pastries, sweets, jams, etc. (z score)	-0.3 (1.1)	0.2 (0.9)	-0.1 (1.1)
Sugar sweetened beverages (z score)	0.1 (1.1)	0.3 (1.2)	-0.3 (0.7)
Fast-food (z score)	0.0 (1.0)	0.3 (1.1)	-0.3 (0.7)
Snacks (z score)	0.1 (1.0)	0.3 (1.1)	-0.3 (0.8)

Data expressed as mean (standard deviation).
